# Protease‐activated receptor 1 drives and maintains ductal cell fates in the premalignant pancreas and ductal adenocarcinoma

**DOI:** 10.1002/1878-0261.12971

**Published:** 2021-05-14

**Authors:** Cansu Tekin, Brendon P. Scicluna, Sophie C. Lodestijn, Kun Shi, Maarten F. Bijlsma, C. Arnold Spek

**Affiliations:** ^1^ Center for Experimental and Molecular Medicine Amsterdam UMC University of Amsterdam The Netherlands; ^2^ Laboratory for Experimental Oncology and Radiobiology Cancer Center Amsterdam Amsterdam UMC University of Amsterdam The Netherlands; ^3^ Oncode Institute Amsterdam The Netherlands

**Keywords:** acinar‐to‐ductal metaplasia, Myc, PDAC, protease‐activated receptor 1

## Abstract

Pancreatic acinar cells have high plasticity and can transdifferentiate into ductal‐like cells. This acinar‐to‐ductal metaplasia (ADM) contributes to tissue maintenance but may also contribute to the premalignant transformation that can eventually progress to pancreatic ductal adenocarcinoma (PDAC). Macrophages are key players in ADM, and macrophage‐secreted matrix metalloproteinase (MMP)‐9 induces ADM through yet unknown mechanisms. As we previously identified MMP9 as a novel agonist of protease‐activated receptor 1 (PAR1), a receptor that is known to orchestrate the cross‐talk between macrophages and tumor cells in PDAC, we here assessed the contribution of PAR1 to pancreatic cell fates. We found that genetic deficiency for PAR1 increases acinar gene expression programs in the healthy pancreas and that PAR1 deficiency limits ductal transdifferentiation in experimental systems for ADM. Moreover, PAR1 silencing in PDAC cells increases acinar marker expression. Changes in PDAC cell lines were associated with a downregulation of known Myc‐target genes, and Myc inhibition mimics PAR1 deficiency in enhancing acinar programs in healthy organoids and PDAC cells. Overall, we identify the PAR1‐Myc axis as a driver of ductal cell fates in premalignant pancreas and PDAC. Moreover, we show that cellular plasticity is not unique to acinar cells and that ductal regeneration into acinar‐like cells is possible even in the context of oncogenic KRAS activation.

## Graphical abstract

Using healthy pancreatic tissue, premalignant transdifferentiation, and ductal carcinoma models, we demonstrated that protease‐activated receptor 1 (PAR1) drives ductal cell fate. PAR1 deficiency prevented ductal transdifferentiation in models for acinar‐to‐ductal metaplasia. We demonstrated that downstream of PAR1 activation, Myc activity drives ductal differentiation programs which goes in line with the known effector functions of Myc in pancreatic ductal adenocarcinoma.

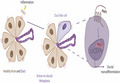

**Abbreviations**
ADM: acinar‐to‐ductal metaplasia
CM: conditioned media
CPA1: carboxypeptidase A1
EGF: epithelial growth factor
GPCR: G protein‐coupled receptor
GSEA: gene set enrichment analysis
KO: knockout
KRT19: keratin 19
MIST1: muscle, intestine, and stomach expression 1
MMP9: matrix metalloproteinase 9
MUC1: mucin 1
NR5A2: nuclear receptor subfamily 5
PanIN: pancreatic intraepithelial neoplasias
PAR1: protease-activated receptor 1
PDAC: pancreatic ductal adenocarcinoma
qPCR: quantitative polymerase chain reaction
shCtrl: shRNA control
shPAR1: shRNA-mediated PAR1 knockdown

## 1 Introduction

Pancreatic ductal adenocarcinoma (PDAC) is the most common type of pancreatic cancer [1]. Five‐year survival rates of PDAC are around 9%, and despite intense research efforts to improve the poor outcome, there has been little improvement in the last decades [1, 2]. Several features of PDAC contribute to the dismal prognosis, including the late stage at which the disease is typically diagnosed [3] and high intrinsic resistance against chemotherapeutics.

In the exocrine pancreas, which facilitates food digestion, two main cell types exist; acinar cells that produce digestive enzymes and epithelial cells that line the ducts through which these enzymes are transported to the duodenum. Despite the strong ductal characteristics and nomenclature of PDAC, the cell of origin of PDAC development remains the subject of debate. In fact, both acinar and ductal cells may give rise to neoplasia leading up to cancerous lesions; nevertheless, acinar cells are reported to be more susceptible to rapid transdifferentiation and neoplasm formation, especially during inflammation (i.e., pancreatitis) [4, 5, 6, 7]. During inflammatory circumstances, the acinar cell state is plastic, and mature acinar cells can de‐differentiate into progenitor cells and subsequently differentiate into duct‐like cells [8]. This acinar‐to‐ductal metaplasia (ADM) contributes to the homeostasis of the pancreas. However, oncogenic activations in acinar cells (typically activating mutations in *KRAS*) and inflammation of the pancreas (pancreatitis) can trigger ADM and give rise to pancreatic intraepithelial neoplasias (PanIN). Additional oncogenic mutations can accumulate in PanIN lesions, which may then progress to PDAC [9]. Studies on genetically modified mouse models have shown TGF‐α expression and KRAS mutations as drivers of ADM and PanIN formation [10]. Additionally, mouse models of cerulein‐induced pancreatitis have shown that inflammation contributes to PDAC development [7]. Furthermore, KRAS mutations can cooperate with the inflammatory cascade to accelerate the formation of malignant lesions [11]. Given the importance of maintaining the appropriate cell fates and lineages for homeostasis and preventing premalignant transformation, acinar differentiation processes are subject to strict regulation. This has typically been attributed to transcription factors with activity in distinct cell populations and (developmental) compartments, but the role of G protein‐coupled receptor (GPCR) signaling known to be important in embryonic development remains underexplored.

Protease‐activated receptor 1 (PAR1) is a GPCR family member that is widely expressed in various tissues in both healthy and disease states. It is activated by proteolytic cleavage of the N‐terminal arm, which liberates a tethered agonist that binds to its activation domain, thereby initiating intracellular signaling. PAR1 was initially described to be activated by the coagulation factor thrombin, but other agonists like MMP1, MMP13, activated protein C, and kallikreins have now been characterized [12]. Increased PAR1 expression is associated with tumor progression and poor prognosis in, among others, breast, lung, and pancreatic cancer [13, 14, 15, 16]. In PDAC, an orthotopic xenograft study revealed that tumor growth is stalled in PAR1‐deficient animals [16]. Interestingly, this reduced tumor growth was accompanied by a significant reduction of macrophage influx into the tumor microenvironment, suggesting a functional link between PAR1, macrophages, and PDAC growth [16].

Macrophages that infiltrate the pancreas are critical players in ADM, and macrophage depletion limits the formation of cerulein‐induced PanIN lesions [17]. Infiltrated macrophages secrete matrix metalloproteinase 9 (MMP9) that targets acinar cells leading to transdifferentiation into ductal cells. This is particularly interesting as we recently identified macrophage‐secreted MMP9 as a novel PAR1 agonist in the setting of PDAC [18]. In the current manuscript, we address the hypothesis that PAR1 plays a more central role in cellular differentiation and that its activation balances acinar and ductal cell fates in the healthy and premalignant pancreas and PDAC.

## 2 Materials and methods

### 2.1 Reagents

Collagenase type IA (Sigma, St. Louis, MO, USA), Collagen I from rat tail (Corning, Corning, NY, USA), trypsin inhibitor (Gibco, Thermo Fisher Scientific, Waltham, MA, USA), IMDM (Gibco, Thermo Fisher Scientific), FBS (#F7524; Sigma), preactivated recombinant MMP9 (Sigma), murine EGF (PeproTech, Rocky Hill, NJ, USA), vorapaxar (Adooq Bioscience, Irvine, CA, USA), p1pal‐12 (palmitate‐RCLSSSAVANRS‐NH2; GL Biochem, Shanghai, China), 10058‐F4 (c‐Myc inhibitor; Selleck Chemicals, Houston, TX, USA), ActinGreen 488 ReadyProbes Reagent (Thermo Fisher Scientific), Dolichos Biflorus Agglutinin (DBA)‐rhodamine (RL‐1032; Vector Laboratories, Burlingame, CA, USA).

### 2.2 Animal studies and ethics approval

C57BL/6 mice (Charles River Laboratories) were housed at the animal facility of the Academic Medical Center of Amsterdam. All mice had access to food and water ad libitum. Institutional Animal Care and Use Committee of Academic Medical Center approved all animal experiments according to protocol number DIX107AA.

### 2.3 Isolation of mouse pancreatic acinar cells

Pancreata from wild‐type and PAR1‐deficient C57BL/6 mice were dissected and washed twice with Hank's buffered saline solution (HBSS; Gibco, Thermo Fisher Scientific). Pancreata were cut with a scalpel to 3‐ to 5‐mm pieces, after which acinar cells were isolated, mostly as described before [19]. In detail, the pancreas pieces were centrifuged at 500 ***g*** for 2 min to remove debris and supernatant. For dissociation of cells, pancreas pieces were next treated with collagenase IA solution (200 U·mL^−1^ collagenase IA, 10 mm HEPES, and 0.25 mg·mL^−1^ trypsin inhibitor in HBSS) for 30 min. Dissociated cells were centrifuged at 500 ***g*** for 2 min and washed twice with wash solution (HBSS supplemented with 5% FBS and 10 mm HEPES) to stop the collagenase reaction. The cell pellet was resuspended in 1 mL seeding medium (IMDM containing 1% FBS, 0.1 mg·mL^−1^ trypsin inhibitor, and 1 µg·mL^−1^ dexamethasone), passed through a 100‐µm cell strainer, collected in 5% FCS‐IMDM medium, seeded in 6‐well plates and incubated for 24 h to separate non‐adherent acinar cells from adherent epithelial and other contaminant cells. The following day, cells in suspension were centrifuged at 500 ***g*** for 2 min and embedded in a collagen mixture (1 mg·mL^−1^ collagen, 1× PBS, 0.1 m HEPES, 0.75% sodium bicarbonate, 0.1 m NaOH) in 6‐well plates with a double 3D layer of 1 mg·mL^−1^ collagen (bottom layer without cells, set prior to seeding).

### 2.4 Viability assessment of acinar spheroids

To assess the viability of collagen‐embedded acini, two different methods were used. First, cells were stained with propidium iodide (PI) at 20 µg·mL^−1^ (final concentration). After 20 min of incubation, phase‐contrast images depicting the explanted acinar cells and Tx‐Red channel images identifying PI‐positive dead cells were taken. As positive control for cell death, 0.5 mm NaN_3_ (sodium azide)‐treated spheroids were used. In our second approach, we performed a resazurin to resofurin reduction (CellTiter Blue) experiment to determine the cells' metabolic activity. In viable cells, resazurin (blue) is converted to resofurin (pink) through reductase activity in the mitochondria. The collagen in which the cells are embedded precluded efficient fluorescence read‐out, and we have used imaging instead to reveal color conversion in the viable cells. In this case, 0.5 mm NaN_3_ (sodium azide)‐treated spheroids were used as a negative control. Images were taken in the color mode of phase‐contrast imaging. In both methods, pictures were taken with the EVOS FL cell imaging system (Thermo Fisher Scientific) at 4×, 10×, and 20× magnification.

### 2.5 Induction of ADM in acinar spheroids

Collagen‐embedded acinar cells were cultured in IMDM containing 1% FBS, 0.1 mg·mL^−1^ trypsin inhibitor, and 1 µg·mL^−1^ dexamethasone. According to a previously described protocol for ADM [17], acinar cells were treated with 50 ng·mL^−1^ murine EGF (PeproTech) or 1 ng·mL^−1^ recombinant MMP9 for 5 days. At the end of the experiment, one batch of cells was visualized with the phase‐contrast channel of the EVOS FL cell imaging system (Thermo Fisher Scientific) at 20× magnification, after which cells were used to isolate RNA. The other batch of cells was fixed with 4% formalin and permeabilized with 0.2% Triton‐X (Sigma). For fluorescence imaging, F‐actin was stained with Alexa488‐conjugated phalloidin (ActinGreen 488 ReadyProbes Reagent; Thermo Fischer Scientific), and ductal structures were stained using rhodamine linked DBA (Dolichos Biflorus Agglutinin; Vector Laboratories). After staining, cells were washed with PBS and imaged with the Yellow Fluorescence (YFP) and Texas‐Red channels of the EVOS FL cell imaging system (Thermo Fisher Scientific) at 20× magnification.

### 2.6 Cell culture

Human PANC‐1, MIA PaCa‐2, and Capan‐2 PDAC cell lines (all obtained from ATCC, Manassas, VA, USA) and primary PDAC cells (i.e., AMC‐PDAC‐096 cells generated from patient‐derived xenografts as described previously [20]) were cultured in high glucose (4.5 g·mL^−1^) containing DMEM (Gibco, Thermo Fisher). All media were supplemented with 10% FBS (#F7524; Sigma), l‐glutamine (2 mm), penicillin (100 units·mL^−1^), and streptomycin (500 μg·mL^−1^) (all Lonza, Basel, Switzerland) according to routine cell culture procedures. Cells were incubated in 5% CO_2_ incubators at 37 °C. All PDAC cell lines were authenticated by STR profiling (Promega PowerPlex, Leiden, the Netherlands) and tested for mycoplasma by PCR monthly.

### 2.7 Immunohistochemistry on paraffin‐embedded material

Orthotopic KP tumors, isolated murine healthy pancreata, and murine organoids were fixed in formalin, embedded in paraffin, and 4‐μm‐thick slides were subsequently deparaffinized, rehydrated, and washed in deionized water. Slides were stained with hematoxylin and eosin (H&E) according to routine procedures. For immunohistochemistry (IHC), endogenous peroxidase activity was quenched with 0.3% hydrogen peroxide for 15 min at room temperature, with antigen retrieval for 10 min at 100 °C in 10 mm sodium citrate buffer, pH 7.4. Slides were blocked for 10 min with Ultra V block (Thermo Fisher Scientific). Primary antibodies against cytokeratin 19 (SAB4501670; Sigma) and amylase (A8273; Sigma) were added (1 : 1600 dilution) for overnight incubation at 4 °C. Slides were subsequently incubated with appropriate HRP‐conjugated goat anti‐rabbit IgG (Brightvision, Immunologic, VWR, Radnor, PA, USA), after which DAB (Brightvision, Immunologic, VWR) staining was used to visualize peroxidase activity. Slides were photographed with a microscope equipped with a digital camera (Leica CTR500; Leica Microsystems, Wetzlar, Germany).

### 2.8 RNA‐Seq

Total RNA was isolated from shCtrl PANC‐1 and *shPAR1* PANC‐1 cells using the NucleoSpin RNA miniprep kit (Macherey Nagel, Düren, Germany). Yield and purity (260 nm : 280 nm) were determined by a Nanodrop ND‐1000 (Thermo Fisher Scientific). The integrity [RNA integrity number (RIN) > 9.0] of the resuspended total RNA was determined by using the RNA Nano Chip Kit on the Bioanalyzer 2100 and the 2100 expert software (Agilent, Amstelveen, the Netherlands). RNA‐sequencing libraries were prepared from 250 ng total RNA using KAPA RNA HyperPrep with RiboErase (HMR) kits (Roche, Basel, Switzerland). Libraries were sequenced using the Illumina HiSeq4000 instrument (Illumina, San Diego, CA, USA) to generate single reads (50 bp). The sequencing depth was approximately 40 million reads per sample. Read quality was assessed by means of the fastqc method (v0.11.5; http://www.bioinformatics.babraham.ac.uk/projects/fastqc/). trimmomatic version 0.36 [21] was used to trim Illumina adapters and poor‐quality bases (trimmomatic parameters: leading = 3, trailing = 3, sliding window = 4 : 15, minimum length = 40). The remaining high‐quality reads were used to align against the Genome Reference Consortium human genome build 38 (GRCh38) [22]. Mapping was performed by hisat2 version 2.1.0 [23] with parameters as default. Count data were generated by means of the HTSeq method [24] and analyzed using the DESeq2 method [25] in the r statistical computing environment (R Core Team [26]). Statistically significant differences were defined by Benjamini and Hochberg‐adjusted probabilities < 0.05. Gene expression extraction was performed via the R2 microarray analysis and visualization platform (http://r2.amc.nl). Sequence libraries are publicly available through the National Center for Biotechnology Information (NCBI) gene expression omnibus (GEO) under the following accession numbers: GSE155010.

### 2.9 Murine healthy ductal organoids

Murine pancreatic ductal organoids were cultured according to the previously described methodology [27]. Organoid media consisted of Advanced DMEM/F‐12 (Gibco, Thermo Fisher Scientific) supplemented with B‐27 supplement (Invitrogen, Carlsbad, CA, USA), *N*‐acetylcysteine (Sigma), nicotinamide (Sigma), gastrin (PeproTech), FGF10 (PeproTech), mEGF (PeproTech), and in‐house produced Noggin and RSPO1 (Table S1). Media were refreshed twice weekly with murine organoid media, and passages were performed weekly with a 1 : 4 ratio with fresh Matrigel (Corning).

### 2.10 Quantitative real‐time PCR

Total RNA was isolated with the NucleoSpin RNA miniprep kit (Macherey Nagel). cDNA was synthesized from DNase‐treated total RNA with M‐MLV‐RT (Promega) and random hexamers (Qiagen, Hilden, Germany). Real‐time quantitative RT‐PCR was performed using the Sensifast SYBR No‐Rox Kit (Bioline, London, UK) on a LightCycler 480 II (Roche). Relative expression levels were calculated using the comparative threshold cycle (dCt) method and normalized for expression of the reference gene TBP. Primer sequences of the analyzed genes are shown in Table S2.

### 2.11 Lentiviral gene silencing

PAR1 silenced KP, PANC‐1, MIA PaCa‐2, and Capan‐2 cells were established with knockdown efficiencies of around 70% for KP, PANC‐1, and Capan‐2 and around 50% for MIA PaCa‐2 cells [28]. The knockdown efficiency of the primary AMC‐PDAC‐096 cell line was around 50% (Fig. S2). For lentiviral silencing of *F2R* in the human PDAC cells, MISSION shRNA library (Sigma‐Aldrich, St. Louis, MO, USA) clone TRCN0000003690 was used, and for the murine KP cells, we used MISSION library clone TRCN0000026806. Clone shc004 was used as control. Lentivirus was produced by transfecting HEK293T cells (ATCC) with 3rd‐generation transfer and packaging plasmids *pVSV*, *pMDL*, and *pRES* using Lipofectamine 2000 (Thermo Fisher Scientific). Forty‐eight and seventy‐two hours after transfection, the supernatant was harvested and 0.45 μm filtered (Millipore, Billerica, MA, USA). Transduced cells were selected with 2 μg·mL^−1^ puromycin (Sigma) for 72 h, after which the transduction efficiency was analyzed by qRT‐PCR.

### 2.12 Gene set enrichment analysis

Datasets used were the tumor expression datasets GSE15471 [29], GSE62452 [30], GSE16515 [31], GSE28735 [32], GSE49149 [33, 34], GSE36924 [33, 35, 36, 37], and TCGA‐PDAC [38]. gsea software (Broad Institute, Cambridge, MA, USA) was downloaded from the Broad Institute website (http://www.broad.mit.edu/gsea/). Acinar and ductal gene signatures were curated based on single‐cell expression profiling of the human pancreas [39]. Expression datasets were compiled with annotated gene names (.gct), samples were segmented for median PAR1/F2R expression (i.e., high and low) as phenotype label files (.cls), and signature sets were assembled (.grp). One thousand permutations were run on the phenotype. Datasets were not collapsed to gene symbols (collapse to gene symbols = false) in the gsea software.

### 2.13 Statistical analysis

Data were presented as mean ± SEM. Statistical analyses were performed using graphpad prism 8.0 (GraphPad Software Inc., La Jolla, CA, USA). Statistically significant differences were considered with a *P*‐value of < 0.05. For further details of the statistical analysis, see figure legends. *P*‐values are indicated by asterisks with *P < 0.05, **P < 0.01, ***P < 0.001, and ****P < 0.0001.

## 3 Results

### 3.1 PAR1 deficiency promotes acinar gene expression programs in the pancreas

**Fig. 1:**
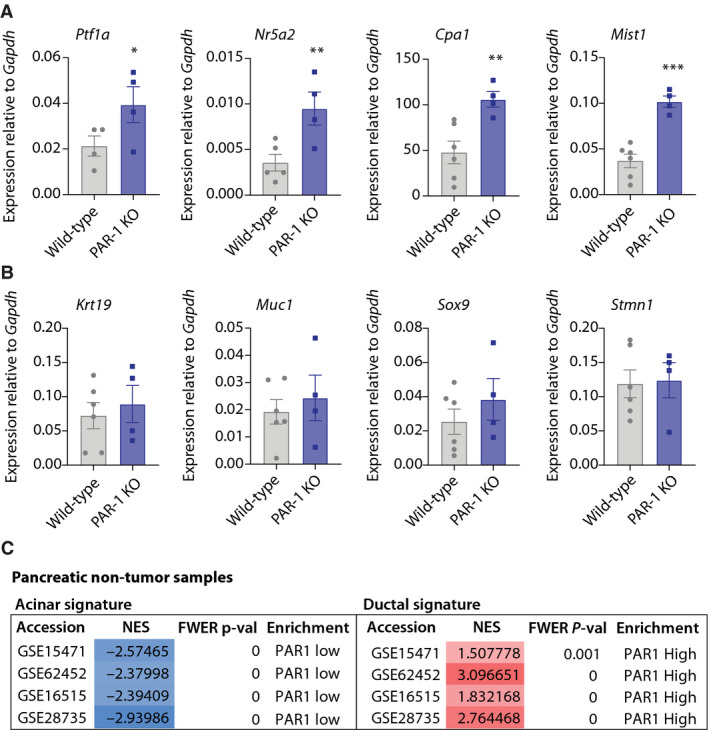
PAR1 deficiency in the pancreas increases acinar‐related gene expression. (A, B) Relative mRNA expression in whole pancreata isolated from wild‐type (gray) and PAR1‐deficient (PAR1‐KO; purple) mice. Shown is the mean ± SEM (wild‐type *n* = 4–6, PAR1‐KO *n* = 4); *P < 0.05; **< 0.01; ***< 0.001, Student's *t*‐test. (A) Acinar‐related marker expression (*Ptf1a*, *Nr5a2*, *Cpa1*, and *Mist1*). Note that samples with poor/missing melt curve were excluded from the analysis. (B) Ductal‐related marker expression (*Krt19*, *Muc1*, *Sox9*) and centroacinar marker expression (*Stmn1*). (C) Gene Set Enrichment Analysis (GSEA) results for human nontumor pancreatic expression sets (dichotomized for median PAR1 expression) with curated acinar (left) and ductal (right) gene signatures. Normalized Enrichment Score (NES) and Family‐Wise Error Rate (FWER) *P*‐values are shown for each tested gene expression set.

In light of the recent identification of macrophage‐secreted MMP9 as a driver of ADM [17] and our data pointing to MMP9 as a novel PAR1 agonist [18], we hypothesized that PAR1 is the receptor that mediates macrophage‐induced ADM. To test this, we first ascertained whether PAR1 deficiency results in altered cell fates in the pancreas. Expression levels of known acinar and ductal markers were measured in pancreata of wild‐type and *F2R*/PAR1‐deficient (PAR1‐KO) mice. Expression levels of the acinar cell marker carboxypeptidase A1 (*Cpa1*) and acinar cell‐fate transcription factors *Ptf1a*, *Nr5a2*, and *Mist1* were strongly increased in PAR1‐deficient pancreata compared with wild‐type controls (Fig. 1A). The expression of ductal and centroacinar markers was not different (Fig. 1B). To corroborate the findings in a human setting, we next assessed the association of PAR1 with acinar and ductal gene sets in human (nontumor) pancreas gene expression datasets. Samples were dichotomized for median PAR1 expression (i.e., PAR1‐high versus PAR1‐low), and gene set enrichment analysis (GSEA) revealed that PAR1 expression negatively correlates with acinar signatures and positively correlates with ductal signatures (Fig. 1C).

### 3.2 ADM requires PAR1 activity *in vitro*

**Fig. 2:**
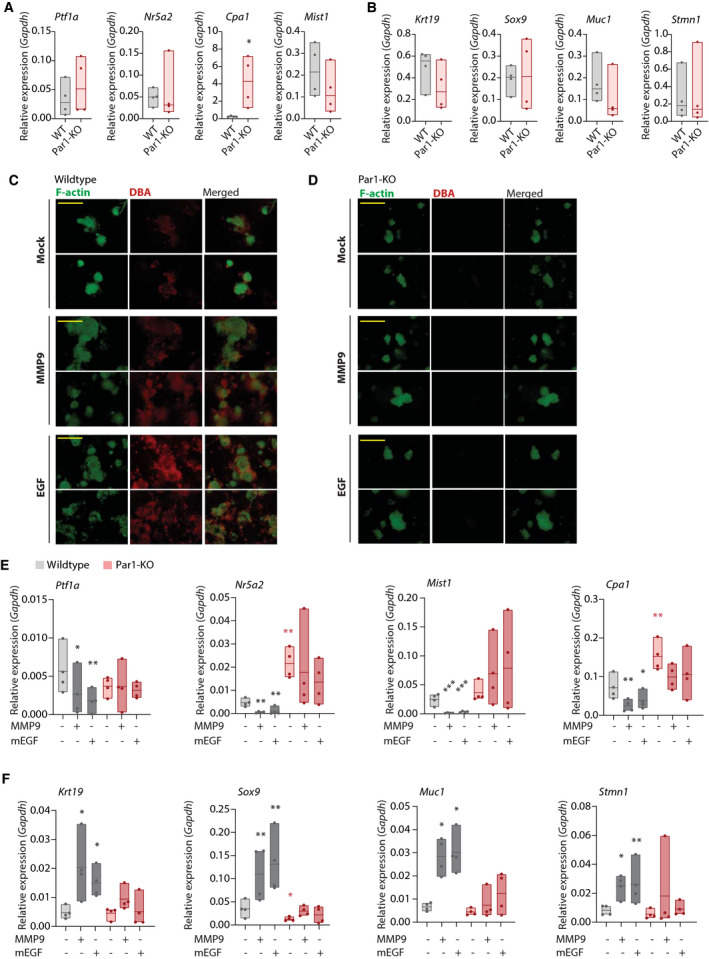
PAR1 deficiency impairs ADM. (A, B) Relative mRNA expression analysis of isolated acinar cells from wild‐type (gray) and PAR1‐deficient (PAR1‐KO; red) mice. Shown is the mean ± SEM (*n* = 4); *P < 0.05, Student's *t*‐test. (A) Acinar‐related marker expression (*Ptf1a*, *Nr5a2*, *Cpa1*, and *Mist1*). (B) Ductal‐related marker expression (*Krt19*, *Muc1*, *Sox9*) and centroacinar marker expression (*Stmn1*). (C, D) Epifluorescence images of 3D collagen‐embedded acinar cells from wild‐type (C) and PAR1‐deficient (D) acinar cells. Acinar cells were treated with DMSO as mock and with 1 ng·mL^−1^ MMP9, or 50 ng·mL^−1^ EGF for 5 days. Scale bars indicate 200 µm. Image acquisition was performed on day 5 at 20× magnification with EVOS FL cell imaging system with YFP and Tx‐Red channels. YFP and Tx‐Red channels were merged on imagej (nih, bethesda, md, usa). (E, F) Relative mRNA expression analysis of 3D collagen‐embedded acinar cells from wild‐type and PAR1‐deficient (PAR1‐KO) pancreas cells. Acinar cells were treated with DMSO (mock) or with 1 ng·mL^−1^ MMP9 and 50 ng·mL^−1^ EGF for 5 days. Shown is the mean ± SEM (*n* = 4); *P < 0.05; **< 0.01; ***< 0.001, One‐way ANOVA. (E) Acinar‐related marker expression (*Ptf1a*, *Nr5a2*, *Cpa1*, and *Mist1*). (F) Ductal‐related marker expression (*Krt19*, *Muc1*, *Sox9*) and centroacinar marker expression (*Stmn1*).

To evaluate the contribution of PAR1 to exocrine pancreatic cell fate, we isolated acinar cells from wild‐type and PAR1‐deficient mice and determined expression levels of acinar (Fig. 2A) and ductal (Fig. 2B) markers. Among all markers tested, only *Cpa1* expression was significantly increased in PAR1‐deficient acinar cells compared with wild‐type cells. This suggests that at steady‐state and in isolated cell populations, PAR1 deficiency does not result in an overt phenotype and indicates that additional interactions (as present *in vivo* such as in Fig. 1C) or cues are required to reveal the contribution of PAR1 to cell fates.

To assess whether PAR1 impacts on ADM, we utilized the same acinar cell isolates using recombinant MMP9 or EGF as positive controls for ADM induction [17]. After 5 days of culturing wild‐type acinar spheroids (viability tested, shown in Fig. S1) in collagen‐embedded cultures, clusters of acinar cells gave rise to duct‐like budding structures (Fig. 2C). The occurrence of these structures markedly increased following the exposure to MMP9 and EGF. To ascertain the ductal identity of these structures, cultures were stained with DBA [40], and additionally, F‐actin was stained to reveal cell mass. Indeed, DBA staining is markedly enhanced in MMP9 and EGF‐treated cultures. Importantly, in Par1‐KO and in vorapaxar‐treated wild‐type spheroids, there was no visible ductal structure formation (also in the presence of MMP9 or EGF; Fig. S2A), and these acinar spheroids were negative for DBA staining (Fig. 2D and Fig. S2B). Subsequent transcript analysis revealed that, after 5 days of ADM induction, expression levels of acinar‐specific transcription factors *Ptf1a*, *Nr5a2*, and *Mist1* and of the acinar marker *Cpa1* were decreased in wild‐type acinar cells, whereas their levels remained unchanged in PAR1‐deficient acinar cells (Fig. 2E). In line with changes in acinar‐related genes, MMP9 and EGF treatment of wild‐type acinar cells led to increased expression levels of the ductal markers *Krt19*, *Muc1,* and *Sox9* and the centroacinar marker *Stmn1* (Fig. 2F). Interestingly, under these culture conditions, the expression of the acinar‐specific markers *Nr5a2* and *Cpa1* and the ductal‐specific transcription factor *Sox9* significantly differed between the two genotypes in the absence of additional ADM cues (Fig. 2E,F). Altogether these data suggest that PAR1 contributes to ductal transdifferentiation of acinar cells in response to macrophage‐secreted MMP9.

### 3.3 PAR1 is associated with ductal characteristics in PDAC

**Fig. 3:**
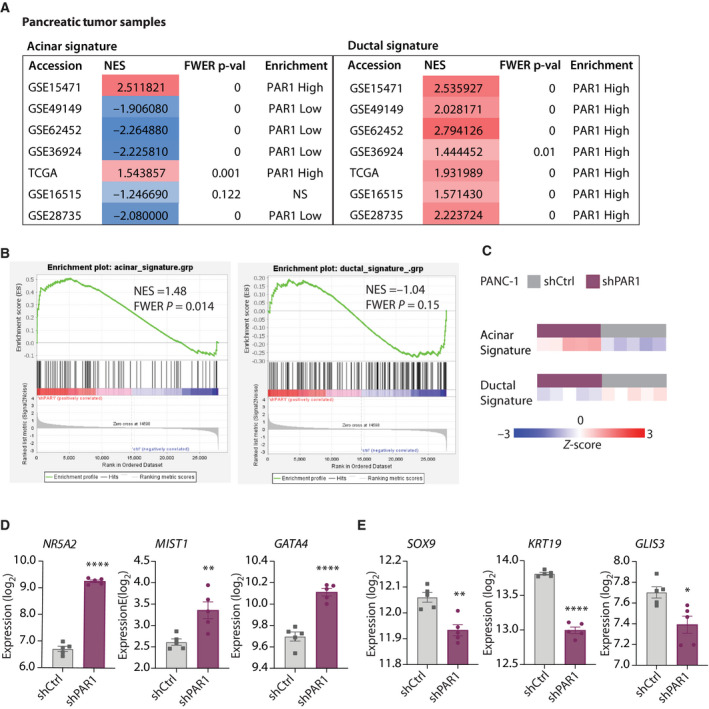
PAR1 expression in ductal tumors is associated with increased ductal marker expression. (A) GSEA results for human PDAC expression sets (dichotomized for median PAR1 expression) with curated acinar (left) and ductal (right) gene signatures. Normalized Enrichment Score (NES) and Family‐Wise Error Rate (FWER) *P*‐value are shown for each tested gene expression set. (B) Enrichment plots (on GSEA) for *shCtrl* PANC‐1 and *shPAR1* PANC‐1 cells with curated acinar (left) and ductal (right) gene signatures. NES and FWER *P*‐values are shown together with the plots. (C) *Z*‐score expression difference analysis on shCtrl PANC‐1 and *shPAR1* PANC‐1 cells with curated acinar (upper) and ductal (lower) gene signatures. Columns indicate data from individual patients. (D, E) Normalized expression levels of acinar‐related genes *NR5A2*, *MIST1,* and *GATA4* (D) and ductal‐related genes *SOX9*, *KRT19*, and *GLIS3* (E) *in shCtrl* and *shPAR1* PANC‐1 cells. Shown is the mean ± SEM (*n* = 5); *P < 0.05, **P < 0.01, ***P < 0.001, and ****P < 0.0001 (Student's *t*‐test).

The observation that ADM appears to depend on PAR1 activity *in vitro* raises the question of whether PAR1 also drives or maintains ductal cell fates in PDAC. To investigate this, we dichotomized eight public PDAC gene expression datasets for PAR1 expression and applied the abovementioned acinar and ductal gene signatures. GSEA analysis showed increased expression of acinar signatures in most PAR1‐low groups, and all sets showed enhanced ductal signatures in the PAR1‐high group (Fig. 3A). To substantiate these findings, we performed RNA‐Seq and GSEA of previously established shRNA‐mediated PAR1‐deficient (*shPAR1*) PANC‐1 cells (knockdown efficiency 70%, see Ref. [28]). Acinar signatures were significantly enhanced in *shPAR1* PANC‐1 cells, and a trend toward a reduced ductal signature was observed in the PAR1‐deficient cells (Fig. 3B,C). Next, we compared expression levels of key acinar‐ and ductal‐related transcription factors and markers (Fig. 3D,E). Expression levels of acinar cell‐fate driving transcription factors *NR5A2*, *MIST1*, and *GATA4* were significantly increased, whereas the ductal transcription factor *SOX9* was markedly decreased in *shPAR1* PANC‐1 cells.

**Fig. 4:**
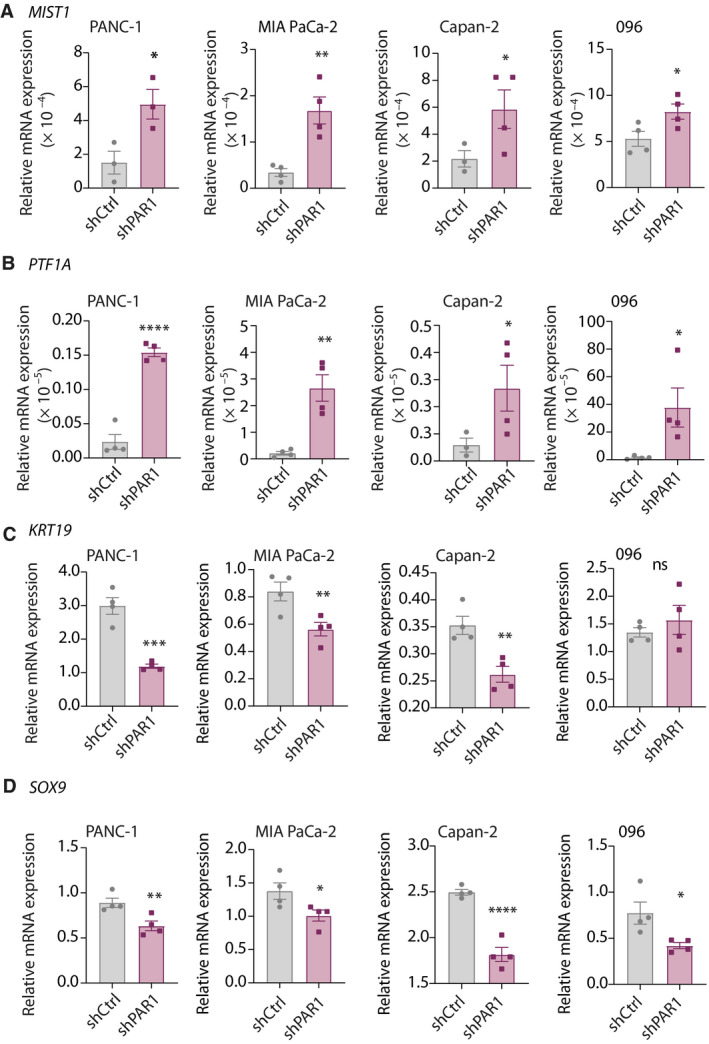
PAR1 downregulation in PDAC cells increases acinar marker expression. (A–D) Relative mRNA expression analysis of *MIST1* (A), *PTF1A* (B), *KRT19* (C), and *SOX9* (D) in shCtrl and *shPAR1* PANC‐1, MIA PaCa‐2, Capan‐2, and 096 PDAC cells. Shown is the mean ± SEM (*n* = 4); *P < 0.05, **P < 0.01, ***P < 0.001, and ****P < 0.0001, (Student's *t*‐test).

This was confirmed by shRNA silencing of PAR1 in two additional routinely used PDAC cell lines (MIA PaCa‐2 and Capan‐2) and one patient‐derived primary line (AMC‐PDAC‐096; 096 in short; knockdown efficiency of PAR1 in the 096 shPAR1 cell line shown in Fig. S3). Transcript analysis of acinar‐specific transcription factors *MIST1* and *PTF1A* showed a significant increase in all PAR1‐deficient PDAC cell lines (Fig. 4A,B). Moreover, expression of the ductal marker *KRT19* and of the transcription factor *SOX9* was significantly decreased in all *shPAR1* cell lines (Fig. 4C,D), with the exception of *KRT19* in the 096 line. These data not only confirm that PAR1 is instrumental in the maintenance of ductal cell fates in cancer but also suggest that loss of its activity can re‐establish acinar cell fates in this context.

**Fig. 5:**
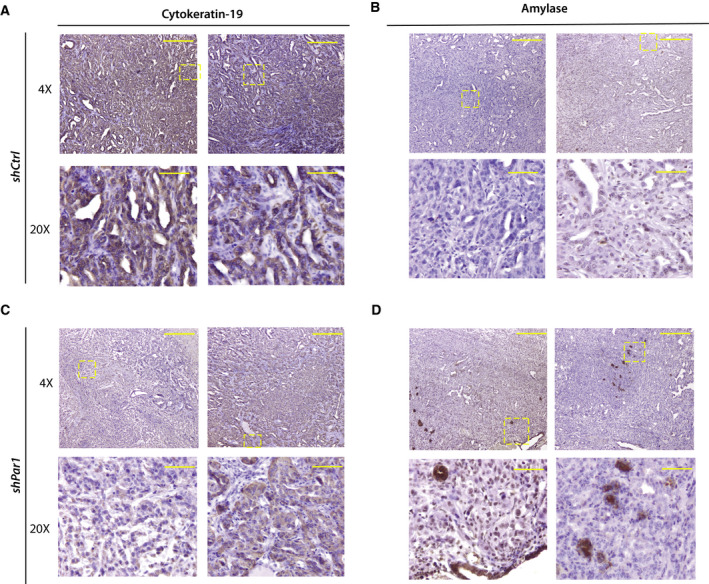
PAR1 silencing in murine PDAC grafts increases acinar marker expression. IHC for cytokeratin 19 (A, C) and amylase (B, D) was performed on orthotopically inoculated shCtrl (A, B) and *shPAR1* (C, D) murine KP tumor sections. Image acquisition was performed at 4× and 20× magnification on a Leica CTR500 microscope. Scale bars indicate on 4× magnification 100 µm and on 20× magnification 500 µm.

We next evaluated KRT19 and amylase expression by IHC in orthotopic tumor cell grafts grown from control or *shPAR1*‐transduced mouse KRAS^G12D^/TRP53^flox/flox^ (KP) tumor cells [28] (Fig. 5). In *shPAR1* tumors, KRT19 expression was decreased compared to control silenced tumors (*shCtrl*). Interestingly, we observed numerous amylase‐positive cell clusters in *shPAR1* tumors, which were (largely) absent in PAR1‐proficient (*shCtrl*) tumors (Fig. 5 with quantification shown in Fig. S4). This confirms that PAR1 not only contributes to ductal cell fates but that its absence partly re‐establishes acinar cell identities.

### 3.4 PAR1 drives ductal cell fates through Myc activity

**Fig. 6:**
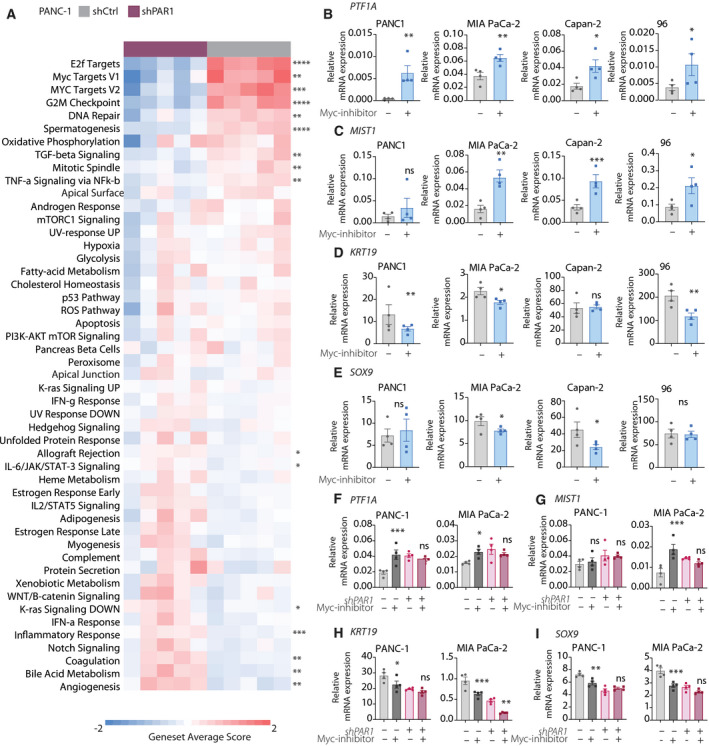
PAR1 drives changes in acinar and ductal‐related gene expression via Myc. (A) Gene Set Map analysis of Hallmark Gene Signatures on shCtrl and *shPAR1* PANC‐1 cells. *P < 0.05, **P < 0.01, ***P < 0.001, and ****P < 0.0001, Student's *t*‐test. (B–E) Relative mRNA expression analysis of *Ptf1a* (B), *Mist1* (C), *Krt19* (D), and *Sox9* (E) in DMSO (control) or 50 µm Myc inhibitor (10058‐F4) treated PANC‐1, MIA PaCa‐2, Capan‐2 and 096 PDAC cells. RNA was collected 48 h after treatment. Shown is the mean ± SEM (*n* = 4); *P < 0.05, **P < 0.01, ***P < 0.001, and ****P < 0.0001, Student's *t*‐test. (F–I) Relative mRNA expression analysis of *PTF1A* (F), *MIST1* (G), *Krt19* (H), and *Sox9* (I) in shCtrl and *shPAR1* PANC‐1 and MIA PaCa‐2 cells after treatment with DMSO (control) or 50 µm Myc inhibitor (10058‐F4). RNA was collected 48 h after treatment. Shown is the mean ± SEM (*n* = 4); *P < 0.05, **P < 0.01, ***P < 0.001, and ****P < 0.0001, Student's *t*‐test.

To elucidate the pathways downstream of PAR1 that govern cell fates, we performed GSEA analysis with the Hallmark Gene Sets [41] on RNA‐Seq data from *shPAR1* and *shCtrl* PANC‐1 cells (Fig. 6A). This analysis revealed a substantial decrease in Myc‐target genes in *shPAR1* cells. This is particularly interesting, given that Myc overexpression in acinar cells has been shown to drive ADM [42]. Consequently, we next assessed whether inhibition of Myc activity impacted on acinar and ductal cell fates. *PTF1A*, *MIST1*, *KRT19*, *SOX9* expression levels in different (i.e., PANC‐1, MIA PaCa‐2, Capan‐2, and 096) PDAC cell lines were measured after treatment with a c‐Myc‐inhibitor (10058‐F4) [43]. This revealed that expression levels of acinar‐specific transcription factors were significantly increased following Myc inhibition (Fig. 6B,C). In line with these changes, Myc inhibition led to a decrease in ductal marker expression (Fig. 6D,E).

To establish whether Myc functions downstream of PAR1 in the maintenance of ductal cell fates, we tested Myc inhibition on control and *shPAR1* PANC‐1 and MIA PaCa‐2 cell lines. In control silenced cell lines, Myc inhibition resulted in decreased ductal and increased acinar marker expression (dark gray bars in Fig. 6F–I). However, Myc inhibition did not affect the expression levels of these markers in *shPAR1* cell lines (compare purple bars to pink bars). Collectively, these findings suggest that PAR1 activity drives ADM and maintains a ductal phenotype through the activity of Myc.

### 3.5 Inhibition of PAR1 and Myc enhances acinar cell fates in murine ductal organoids

**Fig. 7:**
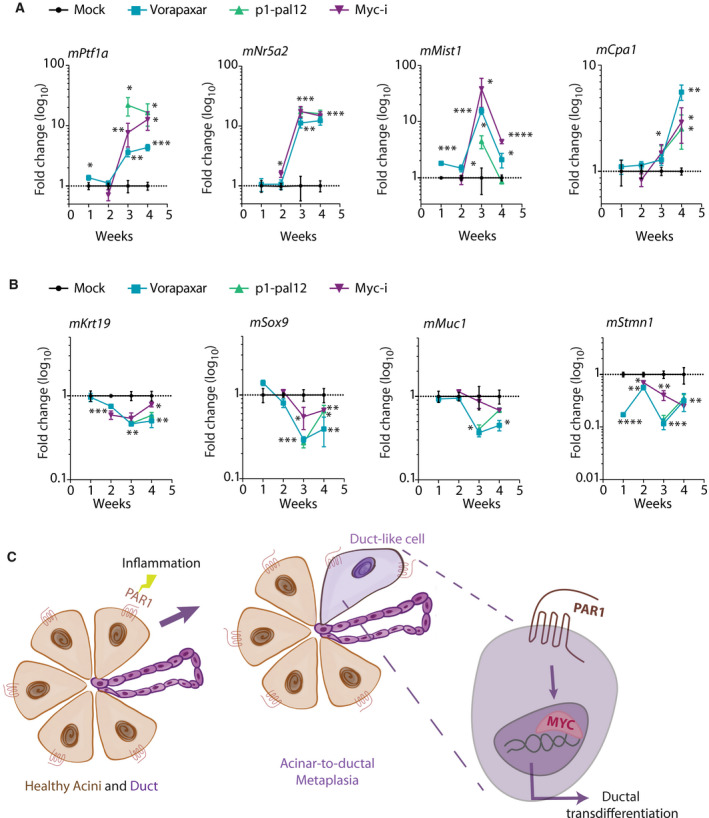
Inhibition of PAR1 and Myc enhances acinar regeneration in healthy murine ductal organoids. (A, B) Relative mRNA expression of (A) Acinar‐related marker expression (*Ptf1a*, *Nr5a2*, *Cpa1*, and *Mist1*). (B) Ductal‐related marker expression (Krt*19*, *Muc1*, *Sox9*) and centroacinar marker expression (*Stmn1*) in murine ductal organoids after treatment with the PAR1 inhibitors vorapaxar (1 µm) and p1pal‐12 (2 µm) or the Myc inhibitor 10058‐F4 (25 µm). DMSO was used as a mock treatment. At each passage (i.e., each week), new inhibitors were added, and RNA was collected from a subset of cells. Fold changes at each time point were calculated based on the expression of the given marker after mock treatment. Shown is the mean ± SEM (*n* = 4); *P < 0.05, **P < 0.01, ***P < 0.001, and ****P < 0.0001, one‐way ANOVA. (A) Acinar‐related marker expression (*Ptf1a*, *Nr5a2*, *Cpa1*, and *Mist1*). (B) Ductal‐related marker expression (*Krt19*, *Muc1*, *Sox9*) and centroacinar marker expression (*Stmn1*). (C) Schematic representation of the PAR1‐Myc axis in ADM.

In tumor contexts, loss or inhibition of PAR1 was able to partially reverse the ductal phenotype of PDAC cells. To further determine whether PAR1 deficiency has a similar impact on ductal maintenance or acinar reprogramming in healthy (nontumor) ductal cells, we utilized murine ductal organoids. Ductal organoids were cultured in the presence of the PAR1 inhibitors vorapaxar or P1‐pal12 and the Myc inhibitor 10058‐F4. The expression of acinar markers *Ptf1a*, *Nr5a2*, *Mist1,* and *Cpa1,* and ductal‐specific markers *Krt19*, *Muc1,* and *Sox9*, and centroacinar marker *Stmn1* were measured. Both PAR1 and c‐Myc inhibition led to an increase in acinar markers during the course of several weeks of treatment (Fig. 7A). Correspondingly, expression levels of ductal markers gradually decreased over time (Fig. 7B). Overall, these results show that PAR1 activation drives ductal differentiation in acinar cells (Fig. 7C) and, more importantly, PAR1 inactivation or Myc inhibition can re‐establish acinar cell identities in ductal cells and shows that such plasticity is not unique to acinar cells.

## 4 Discussion

Transdifferentiation of acinar cells to ductal cells is thought to contribute to tissue regeneration after injury in the pancreas [17, 44]. However, aberrant transdifferentiation of acinar cells into ductal cells during ADM is considered a prerequisite for malignant transformation [45]. ADM critically depends on macrophage‐secreted MMP9 and subsequent NF‐κB activation in acinar cells [17]. However, the cellular receptor on acinar cells through which MMP9 activates the NF‐κB pathway and subsequent ADM has not yet been identified. In the current manuscript, we addressed the hypothesis that PAR1 acts as a key cellular receptor driving cell fate programs in the exocrine pancreas. This hypothesis is based on previous work that highlights PAR1 as the orchestrating receptor of macrophage–tumor cell interactions in PDAC and on the fact that MMP9 has recently been identified as a novel PAR1 agonist [18]. In line with former findings and with our current hypothesis, we show that genetic ablation of PAR1 increases acinar‐related gene expression in the healthy pancreas and that PAR1 deficiency limits ductal transdifferentiation in experimental models for ADM. Moreover, silencing PAR1 in PDAC cells re‐establishes acinar cell identities in these ductal cells. Overall, here we identify PAR1 as a novel cellular receptor to orchestrate ADM, and we show that PAR1 governs cellular identity in the (pre)malignant transformed pancreas.

Acinar cells are thought to have the highest plasticity of all pancreatic cell types and, indeed, rather easily de‐differentiate into a progenitor phenotype, after which they further differentiate into duct‐like cells. Acinar (de)differentiation under benign conditions is reversible, but oncogenic KRAS activation renders this mechanism irreversible and thereby drives ductal metaplasia [8, 46]. KRAS silences the acinar transcription factors MIST1 and PTF1A while it concurrently induces expression of the ductal transcription factor SOX9 [17, 47]. Silencing the acinar transcription factors predisposes acinar cells to ductal differentiation [48] and accelerates ADM [49]. In the current manuscript, we identify PAR1 as a novel player in acinar (de)differentiation in health and disease. We show that PAR1 inactivation leads to increased expression levels of PTF1A and MIST1, whereas expression levels of SOX9 and KRT19 decrease. Importantly, this PAR1‐dependent shift toward an acinar phenotype is not only evident in healthy acinar cells, as PAR1 silencing also increases acinar gene expression in cells from established PDAC. This suggests that also ductal cells are plastic and able to regenerate into acinar‐like cells, even in the presence of oncogenic KRAS activation. Based on these findings, we suggest that PAR1 is critical in ADM and that PAR1 inhibition may prevent malignant progression.

An intriguing finding in our manuscript is the identification of Myc as a downstream factor of the PAR1‐driven cellular differentiation program. Myc inhibition in PDAC cells mimics PAR1 inhibition in re‐activating the expression of acinar transcription factors (PTF1A, MIST1, and NR5A2). Interestingly, Myc activation is known to drive acinar transdifferentiation. Indeed, acinar‐specific Myc overexpression has been described to enhance ductal metaplasia and induce the formation of neoplasms that display both acinar‐like neoplastic cells and duct‐like neoplastic cells [42]. In addition to affecting acinar–ductal tissue homeostasis, our data show that Myc inhibition leads to the re‐expression of acinar‐specific genes in both PDAC and healthy ductal cells. Surprisingly, acinar cell transdifferentiation has not been reported in the range of ductal plasticity in the pancreas [46]. Our findings here suggest that the PAR1‐Myc axis may be a key driver that prevents transdifferentiation of ductal cells back into acinar‐like cells.

Another interesting, and at first glance, surprising finding of our study is that PAR1 deficiency (either by genetic ablation or by pharmacological inhibition) also limits ADM formation by EGF. However, PAR1 is known to transactivate the epidermal growth factor receptor (EGFR) in various experimental contexts. For instance, PAR1 transactivates EGFR in invasive breast carcinoma, thereby promoting cellular invasion [50]. PAR1‐dependent intracellular phosphorylation of EGFR also induces proliferation of vascular smooth muscle cells [51], whereas PAR1 also promotes human colon cancer proliferation through EGFR transactivation [52]. Our data underscore the importance of EGFR–PAR1 cross‐talk in cell fate decisions.

PAR1 expression levels are known to correlate with cancer progression and overall survival in different tumor types of epithelial origin [53, 54]. Consistent with such clinical data suggesting a tumor‐promoting role of PAR1, experimental studies provide solid evidence for PAR1 as a driver of cancer progression. PAR1 induces proliferation, migration, and invasion of cancer cells in *in vitro* experiments [54, 55], whereas tumor cell‐specific PAR1 overexpression drives tumor growth in preclinical animal models of breast and prostate cancer [56]. Inline, inhibition of tumor cell PAR1 [57, 58], stromal PAR1 depletion [16, 59], or pharmacological PAR1 inhibition [14] consistently suppresses tumor growth in experimental animal models. In the current manuscript, we extend this notion by showing that PAR1 not only drives tumor growth but also contributes to the premalignant stages of PDAC. Of note, this effect of PAR1 may be context‐dependent and could be specific for PDAC. Indeed, an elegant prior study showed that PAR1 deficiency in Transgenic Adenocarcinoma of the Mouse Prostate (TRAMP) mice results in diminished apoptosis in transformed epithelia with subsequent larger and more aggressive prostate tumors [60]. Similarly, PAR1 deficiency on the adenomatous polyposis coli mutant (APC Min) background resulted in more and larger adenomas suggesting that PAR1 limits premalignant transformation in prostate and intestinal tumors. In breast cancer, PAR1 deficiency does not seem to modify spontaneous tumor formation [61], further strengthening the notion that PAR1 plays a context‐dependent role in premalignant transformation.

## 5 Conclusion

A picture emerges in which PAR1 is a cellular receptor that governs ductal cell identity. Its perturbation reveals remarkable cellular plasticity in ductal cells. Furthermore, PAR1 represses acinar transcriptional programs to allow regeneration after injury. PAR1 activates the Myc pathway that enhances ADM and locks differentiated acinar cells in a ductal cell state. Downregulating the PAR1‐Myc axis, in part, destabilizes the ductal phenotype leading to the transdifferentiation of PDAC cells. Overall, we identify the PAR1‐Myc axis as a driver of ductal cell fates in healthy, premalignant pancreas and PDAC.

## Conflict of interest

The authors declare no conflict of interest. MFB has received research funding from Celgene and has acted as a consultant for Servier. These parties were not involved in the drafting of this manuscript.

## Author contributions

CT, MFB, and CAS designed the study, interpreted the data, and wrote the manuscript. CT, KS, SCL performed the experiments, and collected data. BPS normalized and analyzed the RNA‐seq data.

## Consent to participate

Not applicable.

## Code availability

Not applicable.

## Supporting information


**Fig. S1.** Viability of 3D collagen‐embedded pancreatic acinar cells. A) Acinar spheroids were treated with mock (DMSO), 1 µM vorapaxar, and 0.5 mM NaN3 on the day of cell seeding. Pictures were taken 4 days after cell seeding/treatment. Propidium iodide (20µg/ml) was supplemented 15 minutes prior to imaging. Phase‐contrast and Tx‐Red (red fluorescence) images are shown at 4X and 20X magnifications. Scale bars indicate 500 µm on 4X, and 200 µm on 20X images. B) Resazurin (blue)‐resofurin (pink) conversion for detection of mitochondrial activity in acinar cultures. Acinar cells were treated as for panel A. 200 µl of CellTiter Blue reagent (resazurin) was added to 1000 µl of culture media and was incubated for 4 hours. Phase‐contrast images are given at 10X and 20X magnifications. Scale bars indicate 400 µm on 10X, and 200 µm on 20X images. All images are taken with the EVOS FL cell imaging system.

**Fig. S2.** PAR1 deficiency and PAR1 inhibition limit ductal differentiation of 3D collagen‐embedded acinar cells. A) Phase‐contrast images of 3D collagen‐embedded acinar cells from wild‐type and PAR1‐deficient acinar cells are shown. Wild‐type (upper panel) or PAR1‐deficient (lower panel) acinar cells were treated with DMSO as mock or with 1µM vorapaxar (only on the wild‐type group), and 1 ng/ml MMP9, or 50 ng/ml EGF for 5 days. Image acquisition was performed on day 5 at 20X magnification with the EVOS FL cell imaging system. Ductal structures are marked with an asterisk (red). B) Fluorescence images of 3D collagen‐embedded acinar cells from wild‐type acinar cells with vorapaxar treatment. Acinar cells were treated with 1 µM vorapaxar and with 1 ng/ml MMP9, or 50 ng/ml EGF for 5 days. Image acquisition was performed on day 5 at 20X magnification with EVOS FL cell imaging system with YFP (F‐actin) and Tx‐Red (DBA) channels. YFP and Tx‐Red channels were merged on ImageJ. Scale bars indicate 200 µm.

**Fig. S3.** shRNA knockdown confirmation of the primary 096 PDAC cell line. Shown mRNA expression for PAR1 (*F2R*) is relative to *TBP*. Shown is the mean±SEM (n=4); * p<0.05, Student's t‐test.

**Fig. S4.** Amylase expression increases in sh*Par1* tumors in the murine orthotopic KP model. Amylase‐positive nuclei were counted on 4X images of 2 independent tumors in both the shCtrl and shPar1 group with the Image J IHC toolbox for DAB staining and nuclei detection (https://imagej.nih.gov/ij/plugins/ihc‐toolbox/index.html). Shown is the mean±SEM (n=2); ** p<0.01, Student's t‐test.

**Table S1.** Culturing media composition and final concentration chart for murine healthy ductal organoids.

**Table S2.** Primer sequences for qPCR analysis of mRNA expression.

**Appendix S1.** Acinar Signature. Acinar cell‐fate related genes used for gene expression analysis.
**Appendix S2.** Ductal Signature. Ductal cell‐fate related genes used for gene expression analysis.

## Data Availability

The data and material are available upon request. RNA‐seq expression profiling is submitted in Gene Expression Omnibus repository (https://www.ncbi.nlm.nih.gov/geo/) under accession code: GSE155010.
