# Supplementary material for: Protease‐activated receptor 1 drives and maintains ductal cell fates in the premalignant pancreas and ductal adenocarcinoma
Source: Mol Oncol. 2021 May 14;15(11):3091–108. doi: 10.1002/1878-0261.12971 (PMC8564660; doi:10.1002/1878-0261.12971)
Supplement: Supplementary file 5 — Table S1. Culturing media composition and final concentration chart for murine healthy ductal organoids. [file MOL2-15-3091-s004.docx]

| **Ingredient** | **Final concentration** |
| --- | --- |
| AdDMEM/F12 (Invitrogen) | Base Media |
| Pen/strep | 1x (10.000 units) |
| Hepes (Sigma, 15630-056) | 5mM |
| Glutamax 10X (35050-038 GIBCO) | 1x |
| B27 supplement (50X) (Invitrogen) | 1x |
| N-acetylcysteine (Sigma) | 1,25 mM |
| Gastrin (Human) (Tocris/Sigma) | 10nM |
| mEGF (Peprotech) | 50ng/ml |
| RSPO1-conditioned media (made in-house) | 10% |
| Noggin-conditioned media (made in-house) | 10% |
| FGF10 (Peprotech) | 100ng/ml |
| Nicotinamide (Sigma) | 10mM |

**Supplementary Table 1.** Culturing media composition and final concentration chart for murine healthy ductal organoids.
