# Supplementary material for: Protease‐activated receptor 1 drives and maintains ductal cell fates in the premalignant pancreas and ductal adenocarcinoma
Source: Mol Oncol. 2021 May 14;15(11):3091–108. doi: 10.1002/1878-0261.12971 (PMC8564660; doi:10.1002/1878-0261.12971)
Supplement: Supplementary file 6 — Table S2. Primer sequences for qPCR analysis of mRNA expression. [file MOL2-15-3091-s009.docx]

| mGAPDH | forward | Mouse | CTCATGACCACAGTCCATGC |
| --- | --- | --- | --- |
|  | reverse | Mouse | CACATTGGGGGTAGGAACAC |
| mMuc1 | forward | Mouse | CCCTATGAGGAGGTTTCGG |
|  | reverse | Mouse | CAGATCAGAGTGCAGGGGT |
| mKrt19 | forward | Mouse | AGGTGAAGATCCGCGACTG |
|  | reverse | Mouse | CGTGTTCTGTCTCAAACTTGG |
| mSox9 | forward | Mouse | CTCTGGAGGCTGCTGAACGAG |
|  | reverse | Mouse | TTGCACGTCGGTTTTGGGAG |
| mCpa1 | forward | Mouse | CAAAACGAATCGCATGTGGC |
|  | reverse | Mouse | CAGGGGTTGCTACTAGCTC |
| mPtf1a | forward | Mouse | TCGAGGCACCCGTTCAC |
|  | reverse | Mouse | TCTCTGGGGTCCACACTT |
| mMist1 | forward | Mouse | TCGAATCCCCAGTTGGAAG |
|  | reverse | Mouse | CTCCGGAGACCCTTTGTCAG |
| mStmn1 | forward | Mouse | GAGCTGATTCTCAGCCCTC |
|  | reverse | Mouse | CGCCTCATGAGACTTGCGT |
| mNr5a2 | forward | Mouse | GGTTTCCTTCCCAAAGTCACA |
|  | reverse | Mouse | CCTGGTGCAGACGCAATAG |

| hTBP | forward | Human | AGACCATTGCACTTCGTGCC |
| --- | --- | --- | --- |
|  | reverse | Human | TGGACTGTTCTTCACTCTTGG |
| hSOX9 | forward | Human | GCTCTGGAGACTTCTGAACG |
|  | reverse | Human | CCGTTCTTCACCGACTTCCT |
| hKRT19 | forward | Human | GGACAAGATTCTTGGTGCCA |
|  | reverse | Human | GTTCCGTCTCAAACTTGGTTC |
| hMUC1 | forward | Human | TCGTAGCCCCTATGAGAAGG |
|  | reverse | Human | CCACTGCTGGGTTTGTGTAA |
| hPTF1A | forward | Human | GTCATCATCTGCCATCGGG |
|  | reverse | Human | AGAGAGTGTCCTGCTAGGG |
| hCPA1 | forward | Human | GCCATTCTCGACACCTTG |
|  | reverse | Human | CCACATGCGATTCGTGCTG |
| hSTMN1 | forward | Human | GGTGCCCCAGGGACCA |
|  | reverse | Human | TAAGGAAAGTCCTGCCGC |
| hNR5A2 | forward | Human | CCCAAGGCCACGAAATTTG |
|  | reverse | Human | GCCCAGCACCAATAGGTGTA |
| hMIST1 | forward | Human | CCCAAGCTCTACCAGCACTA |
|  | reverse | Human | CTCTCGGAAGCTGTGGATC |
| hPAR1 | forward | Human | GCAGGCCAGAATCAAAAGCAACAAATGC |
|  | reverse | Human | TCCTCATCCTCCCAAAATGGTTCA |

**Supplementary Table 2**. Primer sequences for qPCR analysis of mRNA expression.
