# Supplementary material for: Protease‐activated receptor 1 drives and maintains ductal cell fates in the premalignant pancreas and ductal adenocarcinoma
Source: Mol Oncol. 2021 May 14;15(11):3091–108. doi: 10.1002/1878-0261.12971 (PMC8564660; doi:10.1002/1878-0261.12971)
Supplement: Supplementary file 7 — Appendix S1. Acinar Signature. Acinar cell‐fate related genes used for gene expression analysis. Appendix S2. Ductal Signature. Ductal cell‐fate related genes used for gene expression analysis. [file MOL2-15-3091-s005.docx]

**Appendix S1.** Acinar Signature. Acinar cell-fate related genes used for gene expression analysis.

**Appendix S2.** Ductal Signature. Ductal cell-fate related genes used for gene expression analysis.
